# Nono‐titanium dioxide exposure during the adolescent period induces neurotoxicities in rats: Ameliorative potential of bergamot essential oil

**DOI:** 10.1002/brb3.2099

**Published:** 2021-03-10

**Authors:** Yonghua Cui, Yi Che, Hongxin Wang

**Affiliations:** ^1^ State Key Laboratory of Food Science and Technology Jiangnan University Wuxi China; ^2^ Medical College Soochow University Suzhou China; ^3^ School of Food Science and Technology Jiangnan University Wuxi China; ^4^ National Engineering Research Center for Functional Food Wuxi China

**Keywords:** anxiety, bergamot essential oil, memory, Nano‐titanium dioxide, neurotoxicity

## Abstract

**Introduction:**

In adolescence, the brain is still maturing, and disorders in maturation may affect the normal development of the brain. Exposure to titanium dioxide nanoparticles (TiO_2_ NPs) has various potential negative effects on the central nervous system. Bergamot essential oil (BEO) has been found to be effective for neuroprotection.

**Methods:**

The rats were injected intraperitoneally with TiO_2_ NPs (20 mg/kg) and/or BEO (200 mg/kg). The endogenous antioxidant state and inflammatory parameters were estimated using ELISA kits, and then the memory ability and anxiety‐like behavior in rats were assessed.

**Results:**

TiO_2_ NPs exposure during the adolescent period induced anxiety‐like behavior, cognitive impairment, neuroinflammation and oxidative damage in hippocampus, and BEO treatment could significantly ameliorate the neurotoxicities induced by TiO_2_ NPs exposure.

**Conclusion:**

Our results suggest that the negative effects of TiO_2_ NPs exposure during the adolescent period on anxiety‐like behavior and cognitive function may be related to oxidative stress and neuroinflammation induced by TiO_2_ NPs exposure. In addition, BEO may ameliorate the neurotoxicities induced by TiO2 NPs exposure in adolescent rats through the antioxidant and anti‐inflammatory activity of BEO.

## Graphical abstract

There are negative effects of TiO2 NPs exposure during adolescence on emotional control and cognitive functions, and these negative effects are associated with oxidative stress and neuroinflammation in hippocampus induced by TiO2 NPs. BEO is effective in neuroprotection and could ameliorate the negative effects induced by TiO2 NPs.

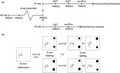

## 1 INTRODUCTION

Nanoparticles are usually defined as particles with a size of less than 100 nanometers. Compared with normal‐sized particles, nanoparticles have higher permeability (Takeuchi et al., 2017). The strong permeability of nanoparticles can be effectively utilized. However, it also poses a potential threat to human health (Orr et al., 2019; Yang et al., 2019). Titanium dioxide nanoparticles (TiO_2_ NPs) are widely used for their whiteness in cosmetics and food, such as sugar‐coated chewing gum, whiten skim milk confectionery, sauces, cakes, pastries, and sunscreens (Chen et al., 2013; Chen & Mao, 2007; Grande & Tucci, 2016; Shi et al., 2013; Warheit & Donner, 2015; Zhang et al., 2015). With the widespread use of this compound, more and more attention has been paid to the potential adverse effects of TiO_2_ NPs on human health. A large number of animal studies have shown that TiO_2_ NPs could accumulate in brain and induce negative impact on brain development (Czajka et al., 2015). Cui et al. (2014) exposed pregnant rats to TiO_2_ NPs and examined the effects of TiO_2_ NPs exposure on brain development in offspring. Results of the study showed that prenatal TiO_2_ NPs exposure impaired the antioxidant status, caused significant oxidative damage to nucleic acids and lipids in the hippocampus of newborn pups, and enhanced the depressive‐like behaviors in adulthood. Another study by Disdier et al. (2017) showed that TiO_2_ NPs exposure in aged rats could induce oxidative stress, brain inflammation, blood‐brain barrier dysfunction and neuronal synaptophysin decrease. In addition, Ze et al. (2014) show that TiO_2_ NPs exposure impaired spatial memory in mice and activated the expression of inflammation cytokines, that is, TNF‐α, IKK1, IKK2, NF‐κB, NF‐κBP52, NF‐κBP65, NIK, and IL‐1β in hippocampus. In accordance with these animal studies, many in vitro studies also showed that TiO_2_ NPs has neurotoxicity and can induce neuroinflammation response and apoptosis. Sheng et al. (2015) showed TiO_2_ NPs treatment resulted in oxidative stress, destabilization of mitochondrial membrane potential (MMP), intracellular Ca^2+^ elevation, and apoptosis in primary cultured hippocampal neurons. In addition, Wu et al. (2010) showed that TiO_2_ NPs treatment could induce dose‐dependent generation of reactive oxygen species (ROS) and neuronal damage in PC12 cells.

Bergamot essential oil (BEO) has been widely used in perfumery and confections for its intense fragrance and freshness (Navarra et al., 2015). In addition, BEO is also used in aromatherapy to improve mood and mild symptoms of stress‐induced disorders (Halcon, 2002; Navarra et al., 2015). With the widespread use of BEO, some biological effects of the BEO have been deciphered by some investigators. BEO has been found to be effective for neuroprotection, anti‐inflammation, and immunomodulation (Corasaniti et al., 2007; Karaca et al., 2007; Navarra et al., 2015). It has been reported that intraperitoneal injection of BEO in rats can reduce the excitatory amino acid efflux and the infarct size of the striatum and motor cortex with middle cerebral artery occlusion (Amantea et al., 2009). Karaca et al. (2007) showed that BEO ameliorated the inflammation activity induced by carrageenan in rats. Meanwhile, it has been proved from a vitro study by Corasaniti et al. (2007) that BEO can prevent the accumulation of intracellular ROS and reduce cell death of human neuroblastoma (SH‐SY5Y) induced by N‐Methyl‐D‐aspartic acid (NMDA).

TiO2 NPs exert potential negative effects on the central nervous system while BEO is effective in neuroprotection and anti‐inflammation. Both BEO and TiO2 NPs are used in modern lifestyle products that adolescents like. In addition, adolescence is a critical period of brain maturation. Hippocampus is an important part of the brain related to emotion control and cognitive function, and it is still in the mature stage in adolescence (Hueston et al., 2017; Romeo et al., 2016; Tottenham & Galvan, 2016). Therefore, it would be interesting to assess the potential effects of TiO_2_ NPs and BEO on the development of hippocampus in adolescents. In the present study, adolescent rats were exposed to TiO_2_ NPs; the endogenous antioxidant state and inflammatory parameters in hippocampus, as well as the memory ability and anxiety‐like behavior of adolescent rats, were evaluated; and the potential ameliorate effects of BEO on TiO_2_ NPs exposure were assessed.

## 2 MATERIALS AND METHODS

### 2.1 Chemicals

TiO_2 _NPs were obtained from Zhejiang Hangzhou Wanjing new material Co, Ltd. (Hangzhou, China). The details of the characterization of TiO_2_ NPs were previously described by Ma et al. (2009). TiO_2_ NPs were suspended in 0.9% sodium chloride at a concentration of 10 mg/ml and treated with ultrasound for more than 60 min before the start of experiments. BEO extracted from bergamot (Citrus medica cv. sarcodactylis), was obtained from Zhejiang Jinshoubao Biotechnology Ltd. The BEO samples were analyzed using GC and confirmed using GC‐MS to identify the major compounds (60.91% limonene, 27.08% γ‐terpinene, 1.71% α‐pinene, 1.70% β‐pinene, 1.58% β‐myrcene, 1.33% cyclohexene). Emulsions of BEO were freshly prepared with soybean oil at a concentration of 100 mg/ml just before the start of experiments.

### 2.2 Animals and treatments

**FIGURE 1:**
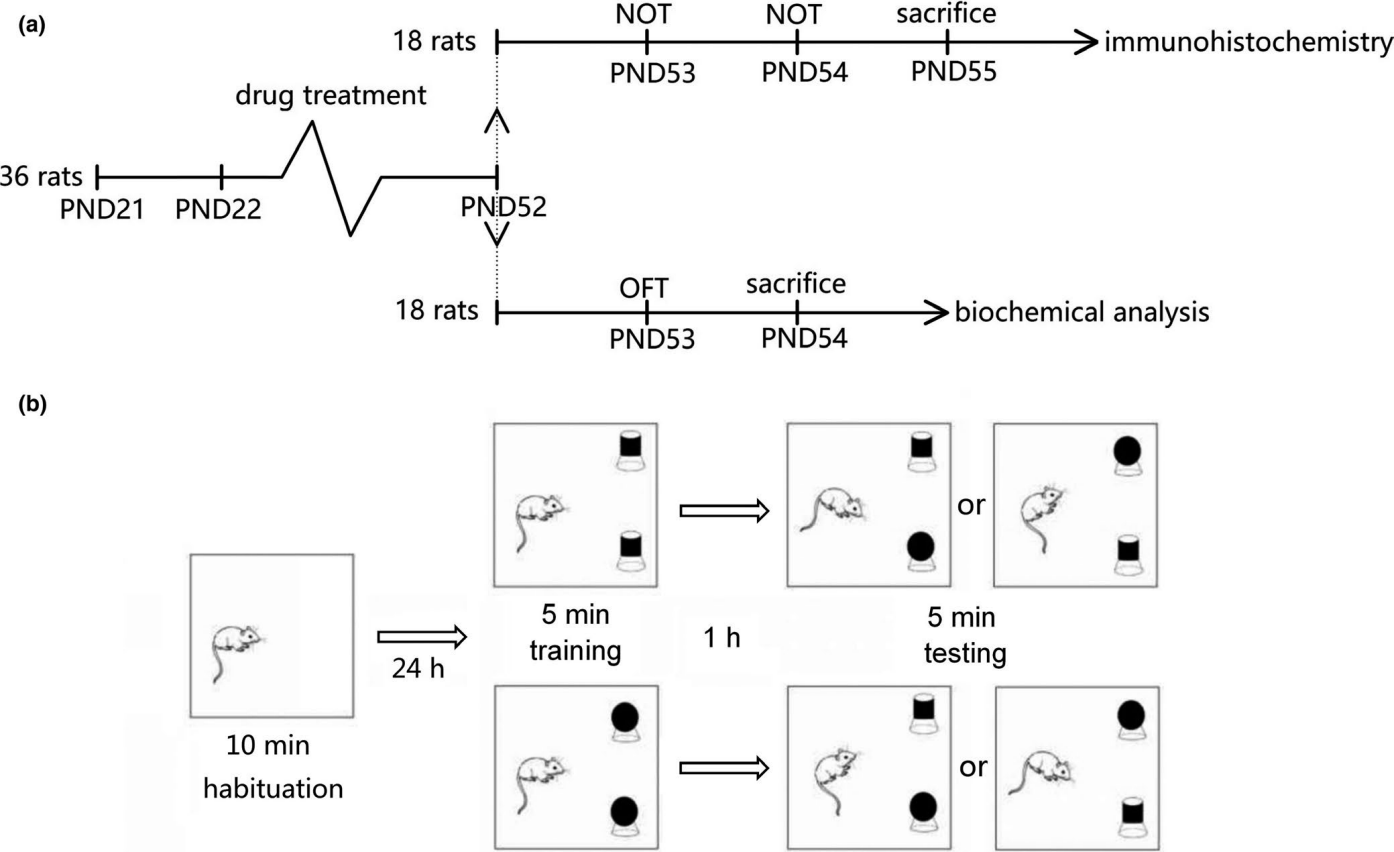
The process of experimental operation. (a) Timeline of the experiment. A total of 36 male Sprague Dawley (*SD*) rats aged PND 21 were used in the experiment. Rats were treated with drug from PND 22 to PND 52. After 31 days of treatment, half of the rats (18 rats) were used to performed the novel object recognition test on PND 53 and PND 54 and then were sacrificed on PND 55 for immunohistochemistry test; the other half of the rats (18 rats) were used to performed the open field test on PND 53 and then were sacrificed on PND 54 for biochemical analysis. PND, postnatal day; OFT, open field test; NOT, novel object recognition test. (b) The experimental procedure of the novel object recognition test. In the training trial, each rat was placed in the square box with two identical objects for 5 min. After 1 hr, the testing trial was conducted, and each rat was placed back into the test box for another 5 min with one of the two identical objects replaced by a novel object. Choice of the objects to be explored and their positions were decided according to a random number table

A total of 36 male Sprague Dawley (*SD*) rats aged postnatal day 21 (PND 21) were provided by the Animal Center of Soochow University. All experiments were conducted in accordance with procedures approved by the Animal Experimental Committee, Soochow University (Approval Number: 2,170,357). The rats were kept in a climate‐controlled colony room at 24℃ on a 12/12 h reverse light/dark cycle with free access to standard food and water. Rats were randomly divided into three groups (*n* = 12 for each group): TiO_2_ NPs group (TiO_2_ NPs + BEO vehicle); BEO group (TiO_2_ NPs + BEO); control group (TiO_2_ NPs vehicle + BEO vehicle). Rats were treated with TiO_2_ NPs (20 mg/kg) (Hu et al., 2010; Younes et al., 2015) and BEO (200 mg/kg) (Gao & Tian, 2012) from PND 22 to PND 52. The TiO_2_ NPs group was injected intraperitoneally once every two days with TiO_2_ NPs (20 mg/kg) and given by gavage once a day with a dose of BEO vehicle. The BEO group was injected intraperitoneally once every two days with TiO_2_ NPs (20 mg/kg) and given by gavage once a day with BEO (200mg/kg). The control group was injected intraperitoneally once every two days with a dose of TiO_2_ NPs vehicle and given by gavage once a day with a dose of BEO vehicle. The concentration of TiO_2_ NPs was 10 mg/ml and the amount of intraperitoneal injection was 0.16–0.6 ml. The concentration of BEO was 100 mg/ml and the gavage volume was 0.16–0.6 ml. After 31 days of TiO_2_ NPs and BEO treatment, half of the rats (18 rats, *n* = 6 for each group) were used to perform the novel object recognition test on PND 53 and PND 54 and then sacrificed on PND 55 for immunohistochemistry test; the other half (18 rats, *n* = 6 for each group) were used to perform the open field test on PND 53 and then sacrificed on PND 54 for biochemical analysis. The timeline of the experiment is shown in Figure 1a.

### 2.3 Novel object recognition test

The novel object recognition test is commonly used to assess memory. After the rats were exposed to TiO_2_ NPs and BEO from PND 22 to PND 52, the memory of rats was evaluated in the novel object recognition test. Eighteen rats (*n* = 6 for each group) were used for the novel object recognition test in the morning of PND 53. The test room was uniformly illuminated by a red light. A square box (100 × 100 × 50 cm) and two kinds of objects (approximately 13 cm high) were used for the novel object recognition test. The shape, color and texture of the two kinds of objects were different. The objects had enough weight and would not be moved by rats. According to the published novel object recognition test protocols (Che et al., 2015; Leger et al. 2013; Lindsay, 2017), the experimental procedure of novel object recognition test included three periods: habituation, training, and testing. Briefly, the habituation trial was performed on PND 53. In the habituation trial, each rat was placed in the empty square box for 10 min. Training and testing were performed on PND 54. In the training trial, each rat was placed in the square box with two identical objects for 5 min. The discrimination index of left and right position preference is measured by the following formula:

Discrimination Index duringtraining=(Tr‐Tl)/(Tr‐Tl)×100%.

Tr stands for the right object and Tl for the left object.

After 1 hr, the testing trial was conducted, and each rat was placed back into the test box for another 5 min with one of the two identical objects replaced by a novel object. The discrimination index of familiar objects and new objects was measured by the following formula:

Discrimination Index duringtraining=(Tn‐Tf)/(Tn+Tf)×100%.

Tn represents the exploration time devoted to the novel object and Tf represents the exploration time devoted to the familiar object.

In order to avoid the influence of natural preference and positions on the results as much as possible, as shown in Figure 1b, choice of the objects to be explored and their positions were decided according to a random number table. Animal behavior in the box was recorded by a video camera. Total time spent to explore the familiar object and the novel object was measured with the video tracking system software (Noldus Information Technology Inc.). After each trial, the behavior box and objects were cleaned thoroughly with 70% ethanol.

### 2.4 Open field test

The open field test is commonly used to assess anxiety‐like behavior in rodents. After the rats were exposed to TiO_2_ NPs and BEO from PND 22 to PND 52, the anxiety‐like behavior of rats was evaluated in the open field test. Eighteen rats (*n* = 6 for each group) were used to perform the open field test in the morning of the PND 53. According to a published open field test protocol (Che et al., 2015). Briefly, the test room was uniformly illuminated by a red light. A square box (100 × 100 × 50 cm) was used for the open field test. The floor was divided into a center area (70 × 70 cm) and a peripheral area. At the beginning of each test, each animal was introduced to the same corner of the arena. Animal behavior in the box was recorded over a period of 5 min by a video camera. The total distance traveled, the distance traveled in the central area, number of rearings and frequency of access to the central area were measured with the video tracking system software (Noldus Information Technology Inc., Leesburg, VA, USA). After each trial, the behavior box was cleaned thoroughly with 70% ethanol.

### 2.5 Biochemical analysis

After open field tests, the 18 rats (*n* = 6 for each group) were used for biochemical analysis according to a published protocol (Cui et al., 2014). Briefly, rats were deeply anesthetized with ketamine (100 mg/kg), the hippocampus of each rat was rapidly isolated on an ice plate, homogenized in ice–cold phosphate buffered saline, and then centrifuged at 10,000 × g for 10 min at 4◦C. The supernatant was used for biochemical analysis. The analysis of malondialdehyde (MDA), catalase activity (CAT), glutathione peroxidase activity (GSH‐PX), and total antioxidant capacity (T‐AOC) were performed using spectrophotometric methods with kits (Nanjing Jiancheng Bioengineering Institute). The IL‐6, IL‐1β, and TNF‐α productions were estimated using ELISA kits (Nanjing Jiancheng Bioengineering Institute).

### 2.6 Immunohistochemistry

After novel object recognition tests, the 18 rats (*n* = 6 for each group) were used to detect the 8‐hydroxy‐deoxyguanosine (8‐OHdG, an oxidatively modified DNA adduct). According to a published immunohistochemistry protocol (Che et al., 2010; Cui et al., 2014), rats were deeply anesthetized with ketamine (100 mg/kg), and perfused with 0.9% saline followed by 4% paraformaldehyde. Then, the brains were placed in 0.01 M phosphate buffer containing 25% sucrose at 4℃ overnight. The brains were cut into 30 μm thick sections. The sections were blocked with 5% normal goat serum in 0.3% Triton X‐100 for 1 hr at room temperature. Then, the sections were incubated with primary antibody (8‐OHdG, QED Bioscience Inc.) at 4 ℃ overnight, incubated with secondary antibody (Alexa‐488, Invitrogen) for 2 hr at room temperature, and mounted using antifade mounting medium. The fluorescence intensity of the hippocampus was counted. This intensity was calculated as follows:

Fluorescence Intensity=Intensity of Hippocampus‐Intensity of the Background.

### 2.7 Data analysis

Statistical analysis was conducted using SPSS 16 (SPSS Inc). All results were presented as mean ± standard error of the mean. Significant differences among groups were analyzed using one‐way ANOVA followed by Newman–Keuls post hoc comparisons test. Statistical significance was set as *p* < .05.

## 3 RESULTS

### 3.1 Effect of BEO treatment on anxiety‐like behavior of rats induced by TiO_2_ NPs in open field test

**FIGURE 2:**
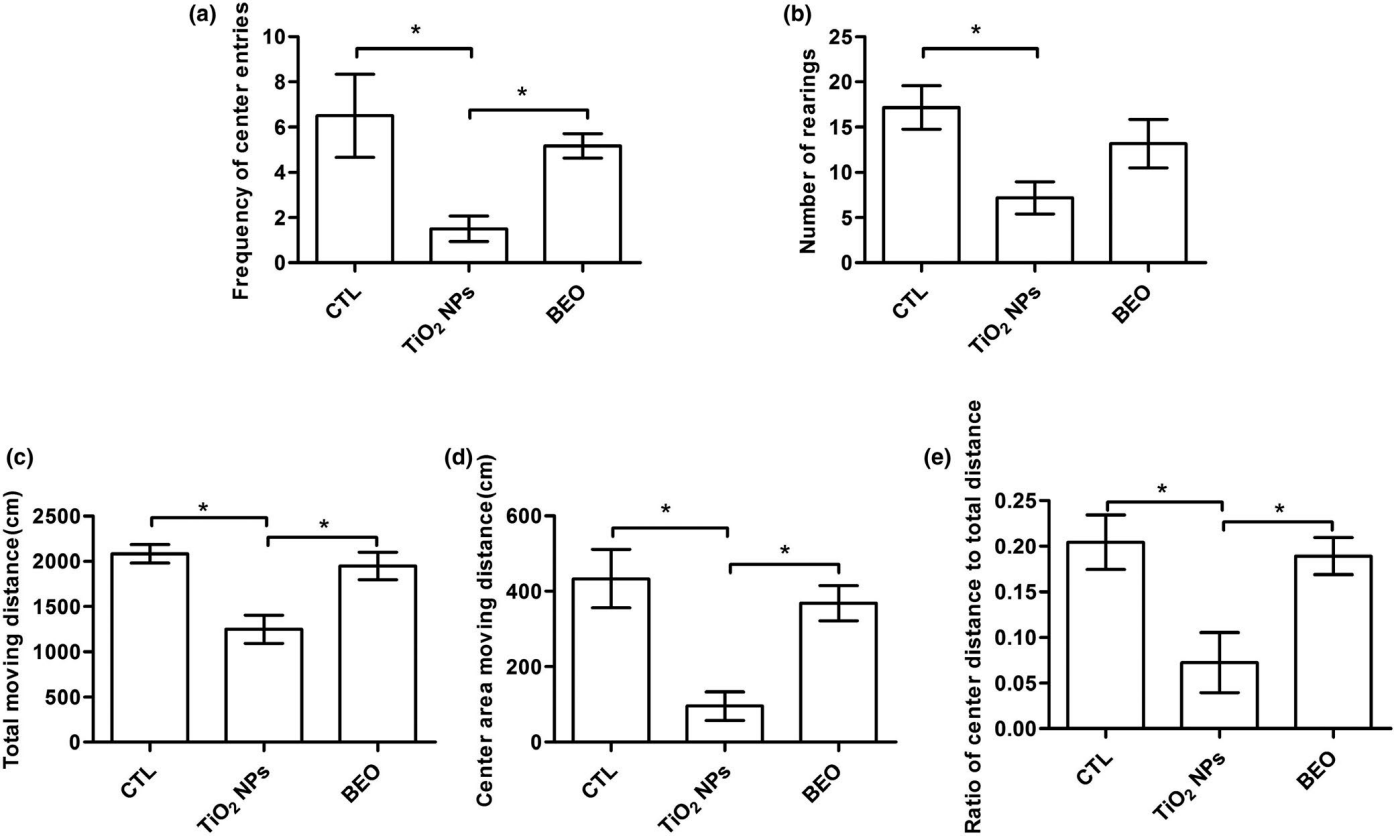
Effect of BEO treatment on anxiety‐like behavior of rats induced by TiO2 NPs in the open field test. (a) the frequency of access to central area in the open field test. (b) the number of rearings in the open field test. (c) the total distance moved in open field test. (d) the distance moved within the center area. (e) the ratio of center distance to the total distance. Results were the mean ± *SEM* (*N* = 6). * *p* < .05; BEO, bergamot essential oil; TiO2 NPs, titanium dioxide nanoparticles; CTL, control; OFT, open field test

As shown in Figure 2, significant differences of the frequency of center entries [*F*(2, 15) = 5.03, *p* < .0212], number of rearings [*F*(2, 15) = 4.72, *p* < .0256], total distance traveled [*F*(2, 15) = 10.42, *p* < .0015], distance traveled within the center area [*F*(2, 15) = 10.09, *p* < .0017] and the ratio of center distance to total distance (center area distance/total distance) [*F*(2, 15) = 6.52, *p* < .0092] among control, TiO_2_ NPs and BEO treatment groups were detected. As shown in Figure 2, compared with the control group, the frequency of center entries, the number of rearings, distance traveled within the center area, and total distance traveled of the TiO_2_ NPs exposure group decreased significantly (*p* < .05). However, the decrease of exploration in the central area might be a result of the reduced total distance moved in the open field. In order to make the judgment of anxiety behavior reliable, the ratio of center distance to total distance was quantified (center area distance/total distance) to determine whether there is a disproportionate reduction in center area inquiry. Compared with the control group, the ratio of center distance to the total distance of the TiO_2_ NPs exposure group decreased significantly (*p* < .05). Compared to TiO_2_ NPs exposure group, BEO treatment group showed a significant increase in the frequency of center entries, distance traveled within the center area, total distance traveled, and the ratio of center distance to total distance (*p* < .05) but not in the number of rearings.

### 3.2 Effect of beo treatment on memory decline of rats induced by TiO2 NPS in the novel object recognition test

**FIGURE 3:**
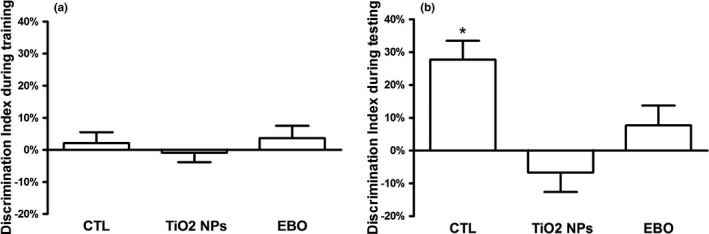
Effect of BEO treatment on memory decline of rats induced by TiO2 NPs in the novel object recognition test. (a) During training, the discrimination index is near zero. Rats have no obvious natural preference for the left or right position. (b) During testing, the control group spent more time exploring the novel object than the familiar one (compared to 0%), but not the TiO2 NPs exposure group and BEO group. Results were the mean ± *SEM* (*N* = 6). * *p* < .05; BEO, bergamot essential oil; TiO2 NPs, titanium dioxide nanoparticles; CTL, control

During training, the time spent to explore the two objects (right and left) were recorded. As shown in Figure 3a, the discrimination index is near zero, indicating that the rats have no left or right position preference. During testing, it is illustrated in Figure 3b that the control group spent more time exploring the novel object than the familiar one (compared to 0%), but not the TiO_2_ NPs exposure group and BEO group. TiO_2_ NPs exposure had significant effects on recognition memory in the novel object recognition test, while BEO treatment did not significantly improve the impairment of recognition memory.

### 3.3 Effect of BEO treatment on oxidative damage in hippocampus of rats induced by TiO_2_ NPS

There were significant differences of the levels of CAT [*F*(2, 15) = 9.73, *p* = .002], GSH‐PX [*F*(2, 15) = 5.62, *p* =.015], T‐AOC [*F*(2, 15) = 7.74, *p* =.004] and MDA [*F* (2, 15) = 5.72, *p* =.014] among control, TiO_2_ NPs and BEO treatment groups (Table 1). TiO_2_ NPs exposure was associated with a decline in the level of CAT, GSH‐PX, and T‐AOC (Table 1). BEO significantly increased the level of CAT (*p* < .05), but not the level of GSH‐PX, and T‐AOC (Table 1). The level of MDA (lipid peroxidation product) was significantly increased in TiO_2_ NPs exposure group compared with the control group (*p* < .05), and BEO significantly decreased the level of MDA (*p* < .05) (Table 1).

**TABLE 1:** Effect of TiO_2_ NPs exposure on antioxidant status in hippocampus of rats

Group	CAT (U/mg protein)	GSH‐PX (U/mg protein)	T‐AOC (U/mg protein)	MDA (nmol/mg protein)
Control	25.89 ± 1.63	35.05 ± 3.03	2.27 ± 0.25	3.98 ± 0.71
TiO_2_ NPs	16.67 ± 1.43^a^	22.84 ± 1.79^a^	1.16 ± 0.12^a^	7.51 ± 0.91^a^
BEO + TiO_2_ NPs	22.62 ± 1.42^b^	30.65 ± 2.83	2.20 ± 0.27	5.02 ± 0.61^b^

Note

Results were the mean ± *SEM* (*N* = 6).

Abbreviations: BEO, bergamot essential oil; CAT, catalase; GSH‐PX, glutathione peroxidase; MDA, malondialdehyde; T‐AOC, total antioxidant capacity; TiO_2_ NPs, Nano‐titanium dioxide.

^a^ *p* < .05, compared with the control group.

^b^ *p* < .05, Compared with the TiO_2_ NPs exposure group.

**FIGURE 4:**
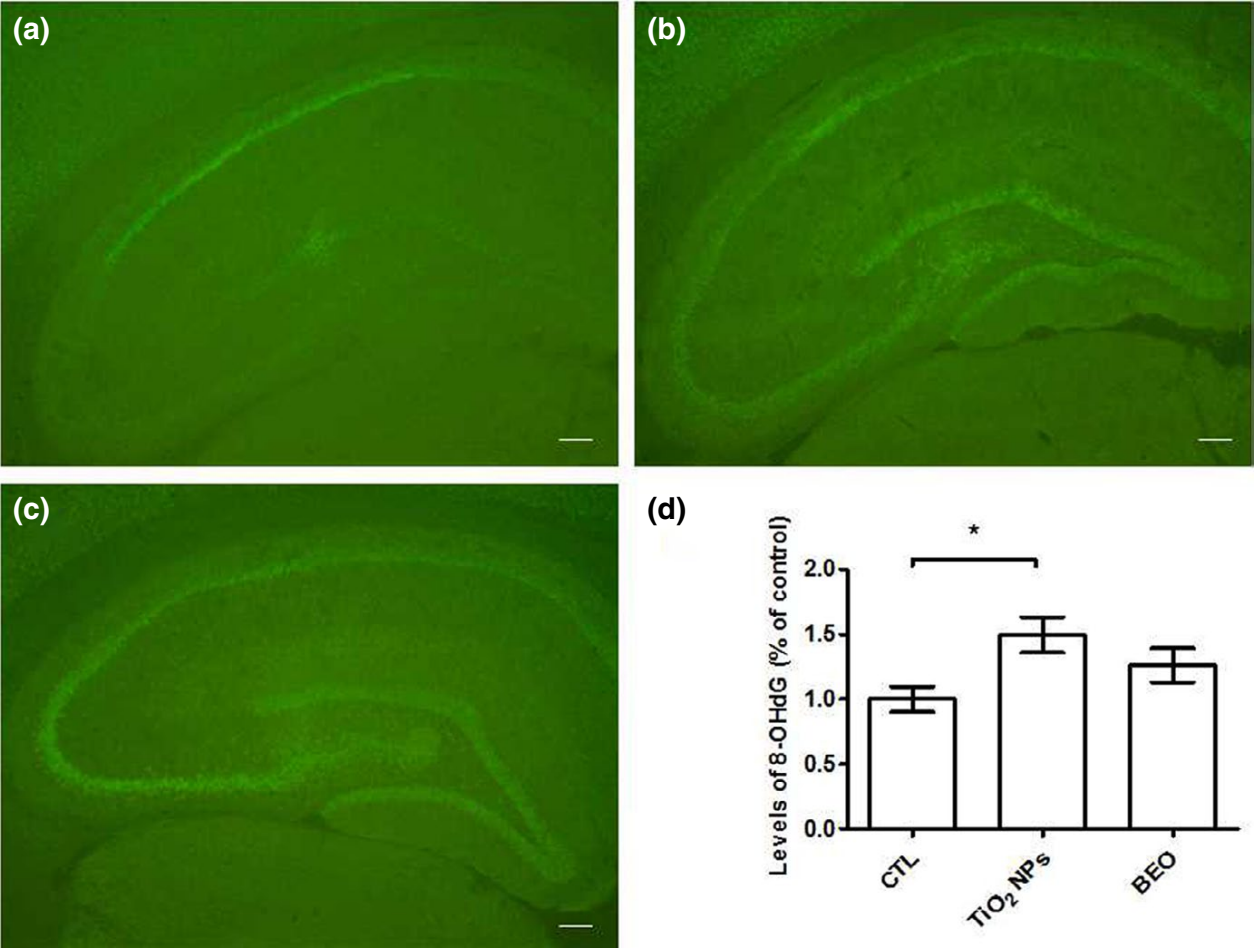
DNA oxidative damage in hippocampus regions of rats. We assessed the DNA damage via immunohistochemical staining with an antibody that recognizes 8‐OHdG in DNA (a) control group. (b) TiO2 NPs exposure group. (c) BEO treatment group. (d) the effect of BEO treatment on DNA oxidative damage in hippocampus of rats induced by TiO2 NPs. Results were the mean ± *SEM* (*N* = 6). * *p* < .05; Scale bars, 250µm; 8‐OHdG, 8‐hydroxy‐deoxyguanosine

The DNA oxidative damage was evaluated by immunohistochemical staining with an antibody that recognizes 8‐OHdG (DNA peroxidation product). There were significant differences of the DNA oxidative damage [*F*(2, 15) = 4.28, *p* = .039] (Figure 4) among control, TiO_2_ NPs and BEO treatment groups. As shown in Figure 4, The DNA oxidative damage was significantly increased in TiO_2_ NPs exposure group compared with the control group (*p* < .05), and BEO did not significantly decrease the DNA oxidative damage.

### 3.4 Effect OF BEO treatment on the increased amounts of cytokines in hippocampus of rats induced by TiO_2_ NPS

As shown in Table 2, significant differences of the levels of IL‐1β [*F*(2, 15) = 4.31, *p* = .032] and IL‐6[*F*(2, 15) = 4.19, *p* = .035] among control, TiO_2_ NPs and BEO treatment groups were detected. No significant difference of the level of TNF‐α was observed [*F*(2, 15) = 2.89, *p* = .086]. Compared with the control group, the levels of IL‐1β and IL‐6 were significantly higher in hippocampus regions of TiO_2_ NPs exposed rats (*p* < .05). Compared to TiO_2_ NPs exposure group, BEO significantly decreased the level of IL‐1β (*p* < .05), but not the levels of IL‐6.

**TABLE 2:** Effect of TiO_2_ NPs exposure on the levels of INF‐α, IL‐1β and IL‐6 in hippocampus of rats

Group	TNF‐α (ng/mg protein)	IL−1β (ng/mg protein)	IL−6 (ng/mg protein)
Control	14.8 ± 1.6	22.3 ± 1.6	14.9 ± 1.4
TiO_2_ NPs	27.7 ± 5.1	30.9 ± 2.1^a^	26.1 ± 3.5^a^
BEO + TiO_2_ NPs	24.1 ± 4.2	24.3 ± 2.7^b^	24.1 ± 3.3^a^

Note

Results were the mean ± *SEM* (*N* = 6).

Abbreviations: BEO, bergamot essential oil; IL‐1β, interleukin‐1 beta; IL‐6, interleukin‐6; TiO_2_ NPs, Nano‐titanium dioxide; TNF‐α, tumor necrosis factor‐alpha.

^a^ *p* < .05, compared with the control group.

^b^ *p* < .05, Compared with the TiO_2_ NPs exposure group.

## 4 DISCUSSION

The open field test is widely used to test animal emotional activity. Exploratory behaviors such as the frequency of access to central area, the distance traveled in the central area and the ratio of center distance to the total distance (center area distance/total distance) are used to demonstrate anxiety‐like behavior (Abelaira et al., 2013; Pollak et al., 2010; Willner et al. 1992). We observed that the TiO_2_ NPs exposure from PND 22 to PND 52 significantly decreased the distance traveled in the central area, the frequency of access to central area and the ratio of center distance to total distance in open field test. The present open field test results show that TiO_2_ NPs exposure during the adolescent period increased anxiety‐like behavior. Moreover, the novel object recognition test based on the natural proclivity of rodents to explore novelty has been used to assess cognitive function (Lindsay, 2017). We observed that TiO_2_ NPs exposure from PND 22 to PND 52 significantly affected the discrimination index of the rats in the novel object recognition test. The present novel object recognition test results show that TiO_2_ NPs exposure during the adolescent period induced cognitive dysfunction. Additionally, in present studies, BEO treatment increased the frequency of access to central area and the ratio of center distance to the total distance in the open field test. The results suggest that BEO treatment can significantly improve the anxiety‐like behavior induced by TiO_2_ NPs exposure. Similarly, a previous study showed that BEO treatment significantly increased the percentage of open arm entries and the percentage time spent in the open arms on the elevated plus‐maze (Saiyudthong & Marsden, 2011). On the other hand, many preclinical studies have shown that BEO can improve stress‐induced anxiety (Lehrner et al., 2000; Ni et al., 2013; Wilkinson et al., 2007). These finding confirmed the anxiolytic‐like properties of BEO (Rombolà et al., 2019; Rombolàet al., 2017; Saiyudthong & Marsden, 2011). Our behavior test findings suggest that TiO_2_ NPs exposure during the adolescent period leads to anxiety‐like behavior and memory impairment, and BEO can improve anxiety‐like behavior.

Adolescence is one of the “critical periods” of brain maturation (Ismail et al., 2017). The plasticity of the nervous system is particularly sensitive to multiple environmental factors in this "critical period" (Dalle & Mabandla, 2018; Schiavone et al., 2015). The age span of adolescence in rats is from PND 21 to PND 59 (Majcher‐Maslanka et al., 2019; McCormick & Mathews, 2010). In the present study, TiO_2_ NPs exposure from PND 22 to PND 52 leads to anxiety‐like behavior and memory impairment. The present results are consistent with the previous studies suggesting that chronic exposure to stressful environmental factors during the adolescent period may lead to emotional disorders and cognitive dysfunction (Romeo et al., 2016; Tottenham & Galvan, 2016).

Hippocampus, which is an important brain area related to emotional control and cognitive functions, is still maturing during the adolescent period. For example, the number of granule cells and the volume of hippocampus are increasing in this period (Hueston et al., 2017; Sousa et al., 1998). Hippocampal neurogenesis during the adolescent period could be altered by acute and long‐term stress (Hueston et al., 2017). TiO_2_ NPs can cross the blood‐brain barrier, increase the generation of ROS (Fujishima et al. 2008), and induce oxidative stress (Gao et al., 2011; Hong et al., 2017). In the present study, we exposed rats to TiO_2_ NPs from PND 22 to PND 52 and evaluated the endogenous antioxidant state and inflammatory parameters in rat hippocampus. Our results show that TiO_2_ NPs exposure during the adolescent period increased the levels of TNF‐α, IL‐1β, and IL‐6, decreased the level of GPx, CAT, and T‐AOC, and increased the lipid oxidative damage and the DNA oxidative damage. The results suggest that TiO_2_ NPs exposure during the adolescent period induced neuroinflammation and oxidative damage in hippocampus. Based on the present and some previous results, we hypothesize that TiO_2_ NPs exposure during the adolescent period induced the generation of ROS (Fujishima et al. 2008); the downregulation of T‐AOC, GSH‐PX, and CAT caused by TiO_2_ NPs exposure during the adolescent period may result in the inability of endogenous antioxidant system to remove excessive ROS; the excessive ROS induced by TiO_2_ NPs exposure during the adolescent period may mediate the neuroinflammation and oxidative damage; and oxidative stress induced by TiO_2_ NPs exposure during the adolescent period may lead to structural and functional damage in the hippocampus, which in turn, impairs the abilities of hippocampus and induces anxiety‐like behavior in open field and memory impairment in novel object recognition test.

Additionally, in the present study, BEO treatment significantly decreased the level of IL‐1β and MDA (*p* <.05), and significantly increased the activity of CAT. The results suggest that BEO ameliorated the neuroinflammation and oxidative damage induced by TiO_2_ NPs exposure during the adolescent period. The anti‐inflammatory and antioxidant activity of BEO or ingredients in BEO (limonene and α‐pinene) have been investigated in previous studies (Wei & Shibamoto, 2007, Kim et al., 2015, Khoshnazar et al., 2019). For example, limonene decreased the level of TNF‐α, IL‐1β, and IL‐6 and the expression of iNOS and COX‐2 in RAW 264.7 macrophage cells (Yoon et al., 2010); α‐pinene exerted antioxidant activities through restoring the activity of superoxide dismutase (SOD), CAT and GSH‐PX, and reducing the concentration of MDA, NO, and IL‐6 in ischemic brain tissue of rats (Khoshnazar et al., 2019); BEO prevented accumulation of intracellular ROS, reduced cell death of human neuroblastoma induced by excessive stimulation of the NMDA (Corasaniti et al., 2007). The BEO used in present study contains 60.91% limonene, 27.08% γ‐terpinene, 1.71% α‐pinene, 1.70% β‐pinene, 1.58% β‐myrcene, 1.33% cyclohexene, some of which may be major contributors to the anti‐inflammatory and antioxidant activity of BEO. In consistent with previous studies, (Corasaniti et al., 2007; Karaca et al., 2007), the present results proved that BEO is effective in neuroprotection.

## 5 CONCLUSION

The present results suggest that there may be negative effects of TiO_2_ NPs exposure during adolescence on emotional control and cognitive functions, and these negative effects are associated with oxidative stress and neuroinflammation in hippocampus induced by TiO_2_ NPs exposure. The negative effect of TiO_2_ NPs exposure during the adolescent period should arouse our attention. On the other hand, BEO is effective in neuroprotection and could ameliorate the negative effects induced by TiO_2_ NPs. However, in the present study, we only studied the effect of BEO treatment and TiO_2_ NPs exposure on male animals, and we could not confirm whether it will have the same effect on female animals. We believe that the biological response effects of TiO_2_ NPs exposure in the adolescent period and the neuroprotective activity of BEO may be complex and need further study.

## CONFLICT OF INTEREST

Authors declare no conflict of interest.

## AUTHOR CONTRIBUTIONS

Yonghua Cui and Yi Che contributed to the experiments. Yonghua Cui and Hongxin Wang contributed to the analyses of the data and the write up of the manuscript.

### PEER REVIEW

The peer review history for this article is available at https://publons.com/publon/10.1002/brb3.2099.

[Correction added on March 20, 2021, after first online publication: Peer review history statement has been added.]

## Supporting information

Fig S1

## Data Availability

The data that support the findings of this study are available from the corresponding author upon reasonable request.
