# Supplementary figures and images for: Nono‐titanium dioxide exposure during the adolescent period induces neurotoxicities in rats: Ameliorative potential of bergamot essential oil
Source: Brain Behav. 2021 Mar 10;11(5):e02099. doi: 10.1002/brb3.2099 (PMC8119869; doi:10.1002/brb3.2099)

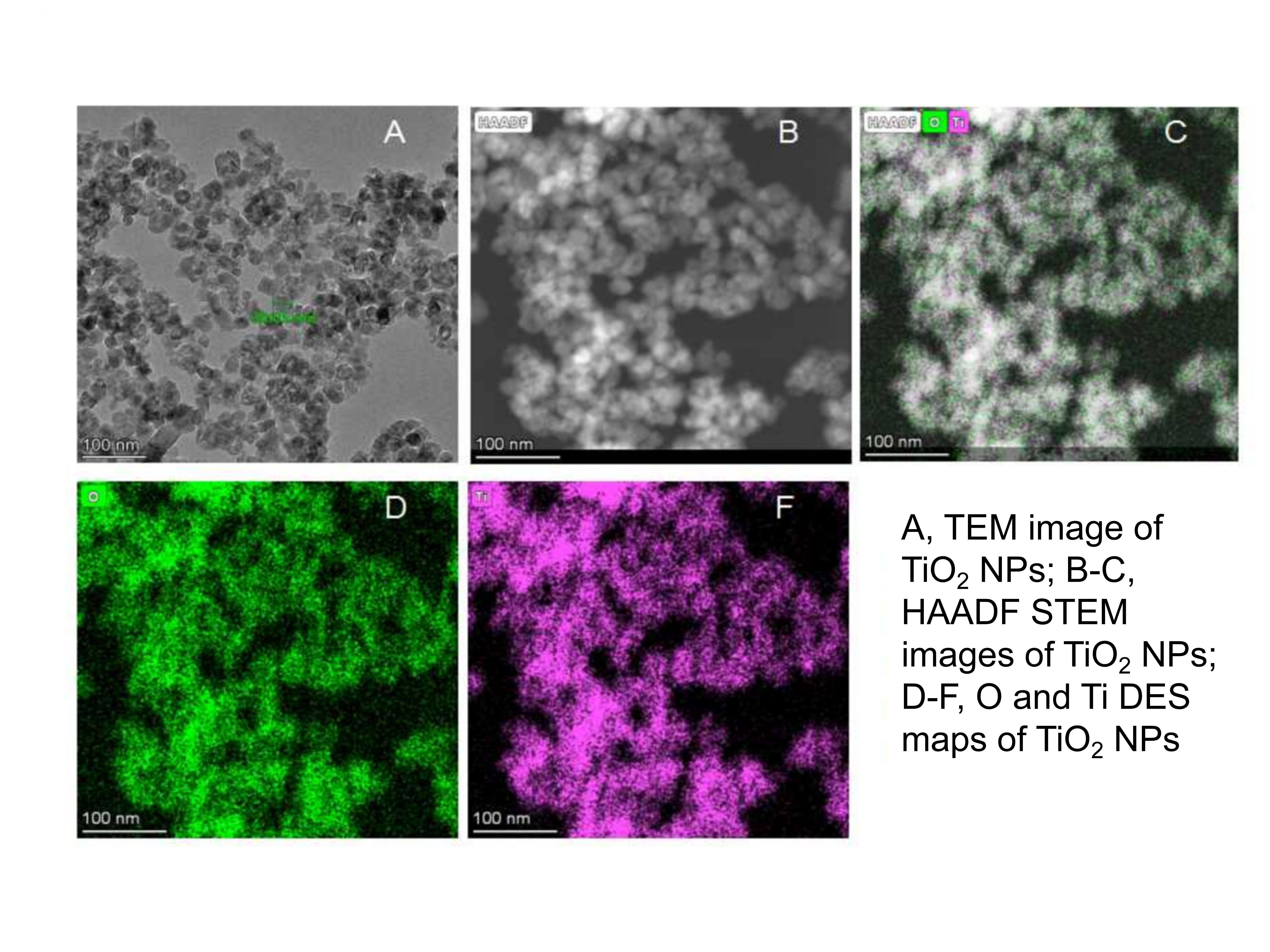

Supplement: Supplementary file 1 — Fig S1 [file BRB3-11-e02099-s001.jpg]
